# High heat tolerance in plants from the Andean highlands: Implications for paramos in a warmer world

**DOI:** 10.1371/journal.pone.0224218

**Published:** 2019-11-06

**Authors:** Indira V. Leon-Garcia, Eloisa Lasso

**Affiliations:** 1 Departamento de Ciencias Biológicas, Universidad de los Andes, Bogotá, Cundinamarca, Colombia; 2 Smithsonian Tropical Research Institute, Panamá, República de Panamá; Chinese Academy of Sciences, CHINA

## Abstract

Tropical plant species are expected to have high heat tolerance reflecting phenotypic adjustments to warm regions or their evolutionary adaptation history. However, tropical highland specialists adapted to the colder temperatures found in the highlands, where short and prostrated vegetation decouples plants from ambient conditions, could exhibit different upper thermal limits than those of their lowland counterparts. Here we evaluated leaf heat tolerance of 21 tropical alpine paramo species to determine: 1) whether species with restricted distribution (i.e., highland specialists) have lower heat tolerance and are more vulnerable to warming than species with widespread distribution; 2) whether different growth forms have different heat tolerance; and 3) whether species height (i.e., microhabitat) influences its heat tolerance. We quantified heat tolerance by evaluating *T*_*50*_, which is the temperature that causes a reduction in 50% of initial *F*_*v*_*/F*_*m*_ values and reflects an irreversible damage to the photosynthetic apparatus. Additionally, we estimated the thermal safety margins as the difference between *T*_*50*_ and the maximum leaf temperature registered for the species. All species presented high *T*_*50*_ values ranging between 45.4°C and 53.9°C, similar to those found for tropical lowland species. Heat tolerance was not correlated with species distributions or plant height, but showed a strong relationship with growth form, with rosettes having the highest heat tolerance. Thermal safety margins ranged from 12.1 to 31.0°C. High heat tolerance and broad thermal safety margins suggest low vulnerability of paramo species to warming as long as plants are capable of regulating the leaf temperature within this threshold. Whether paramo plants would be able to regulate leaf temperature if drought episodes become more frequent and transpirational cooling is compromised is the next question that needs to be answered.

## Introduction

Tropical high elevation Andean ecosystems, locally known as paramos, are said to be particularly vulnerable to climate change due to the large percentage of endemic biota with narrow distributions that are sometimes restricted to a single mountain range [1–4]. Species with narrow distribution ranges are expected to have narrow habitat tolerances. Therefore, high rates of species loss and turnover are expected with climate warming in paramos ecosystems [4], with the somber expectation that 60% of the flora could be lost or critically endangered by 2050 [5]. Most of these estimates are built on broad scale isotherm-based models that fail to account for the complex patterns of temperature in alpine landscapes [6–8] and are therefore likely overestimating the extinction of species in alpine sites [7]. Responses to climate change can differ between species; those with great migration capacities could be able to track the displacement of their niche, while those with high heat tolerance could be able to cope with new conditions [9].

Heat tolerance of tropical alpine species has been scarcely studied, in contrast to temperate alpine species where broad thermal tolerance and significant acclimation potential to warming have been reported [10–13]. Considering that threshold of upper temperature in paramos has been rarely studied, evaluating heat tolerance in this ecosystem could help assess the vulnerability of paramos vegetation and improve predictions on the impact of warming on tropical biodiversity [14,15].

Evaluating heat tolerance has proven to be a useful tool for studying species vulnerability to warming [10–12,16–18] since it gives an insight into the plant’s ability to tolerate heat stress and into the damage that can be caused to all membranes including those in the photosynthetic apparatus [19,20]. Heat stress can affect both biochemical and physiological processes [21], and when critical temperatures are reached membrane breakage, electrolyte leakage and leaf necrosis occurs [11,17,22]. One of the most heat sensitive parts of the photosynthetic apparatus is the photosystem II (PSII), the protein complex involved in the oxidation of water and the initiation of electron transport [11]. When PSII is exposed to stress, its capability for processing light on photochemical reactions decreases and the unused energy is then re-emitted as fluorescence. Therefore, different fluorescence parameters are used as indicators of photoinhibition, *F*_*v*_*/F*_*m*_ being one of the most reliable and commonly used. The heat tolerance of a plant can be estimated by recording the irreversible decrease in *F*_*v*_*/F*_*m*_, an indication of a reduction in the maximum efficiency of PSII [17,23] in response to changing temperature. The critical temperature or *T*_*50*_ is a common estimate of heat tolerance and indicates the temperature that causes a 50% reduction in *F*_*v*_*/F*_*m*_, which typically occurs when the damage to PSII is irreparable, leaves become necrotic and net carbon assimilation declines [11,17,22].

Heat tolerance of tropical forest plants is considered a conserved trait with values around 50°C in most studied species, reflecting a general threshold of physiological tolerance to heat for C_3_ plants [24,25], and indicating no correlation between environmental temperature and critical limits to heat [24]. However, scarce data exists on the heat tolerance for high elevation tropical species living above 3,000 meters and regularly exposed to temperatures below 10°C [26]. At this elevation, surveys of leaf and plant temperatures have shown that leaf and air temperatures are not in equilibrium, especially in prostrated forms [27–29]. We would therefore expect heat tolerance to respond to the degree of decoupling the species can achieve, which will be mostly related to traits such as growth form, height and leaf morphology [29,30].

Most of our knowledge of heat tolerance at high elevations comes from temperate alpine plants, whose heat tolerance is higher than what would be predicted from their thermal environment [30]. Studies from alpine sites indicate that heat tolerance is correlated with functional traits and growth form; species with conservative resource strategies such as low specific leaf area (SLA) and long lived leaves had higher values of heat tolerance [21,25,31]. Furthermore, low-stature rosettes and cushion plants tolerate higher temperatures than forbs, and shrubs [12]. These studies suggest that thermal tolerance is strongly influenced by the temperatures experienced by the plant [12,32], which is influenced by height, leaf morphology and growth form [29,30], so that when leaf temperatures exceed a specific threshold, heat tolerance tends to raise [33]. However, high values of heat tolerance have also been found in plants with low leaf temperatures, so the relationship between heat tolerance and leaf temperature remains unclear [12]. From the high-elevation tropics, most of our knowledge on thermal tolerance comes from studies focused on the lower thermal limits that cause freezing injuries [34–39], while the heat tolerance of tropical highland species is still poorly understood.

In this study, we evaluated the heat tolerance and the thermal safety margins of 21 common paramo species from the oriental range of the Colombian Andes. The thermal safety margin (TSM) is considered a good estimate of species vulnerability to climate change and their physiological capacity to deal with, and be protected from, critical temperatures [40]. TSM is often calculated based on air temperatures rather than leaf temperature [41,42], which could have low biological meaning when leaf temperature is decoupled from atmospheric conditions, a common phenomenon in tropical alpine species [28,29,43,44]. Here we calculated the TSM using the maximum daily leaf temperature (*T*_*max*_) recorded with thermal images. In order to identify possible proxies for heat tolerance, we selected species with different growth forms, heights and range distribution to evaluate the relationship between those traits and species heat tolerance. We hypothesized that: 1) species with widespread distribution in elevation that are adapted to broader thermal niches will have higher heat tolerance than species restricted in their elevation to paramos (highland specialists); 2) shorter plants will have higher heat tolerance than taller plants, since plants growing closer to the ground are exposed to more extreme thermal microhabitats [29,34]; and 3) rosette forms, usually pubescent in paramo, will have higher upper thermal tolerance than other growth forms because their insulation structures reduce heat transfer from the leaf to the air. This is a valuable strategy during freezing periods, but it is also a trait that could result in overheated leaves on sunny days [43] and would therefore require these species to have higher heat tolerance.

## Materials and methods

The authors hereby state that all the collection of plant material made on the privately owned land "Parque Ecológico Matarredonda", were under the approval of "Permiso Marco de Recolección de Especímenes de Especies Silvestres de la Diversidad Biológica con fines de Investigación Científica No Comercial" from the "Autoridad Nacional de Licencias Ambientales (ANLA)" with the permit number: IBD0359—Res 1177 of October 9, 2014.

### Study site

Measurements were taken on 21 paramo species from the Parque Ecológico Matarredonda (4°33’38.1” N and 74°0’7.3” W) located in the oriental range of the Colombian Andes. Elevation at this park ranges from 3,100 to 3,600 m.a.s.l. The mean annual temperature (MAT) at the study site is 8.8°C [26]; mean annual precipitation is 1,178 mm and the mean relative humidity is 88% [45]. To better describe the actual climate experienced by plants (microclimate) at the study site, we recorded air temperature at 30 cm above the soil surface every 30 minutes from December 2017 to December 2018 using an automatic recording system consisting of an EM-50 data logger (Decagon Devices, Inc., Pullman, WA) connected to a VP-4 humidity and temperature sensor with a radiation shield installed (Decagon Devices, Inc., Pullman, WA).

### Growth form, height, and elevation range

To evaluate proxies for heat tolerance, 21 species were selected with different elevation distribution ranges, growth forms (shrubs, forbs, grasses and rosettes) and heights (Table 1). The elevation range (the upper and lower limits of the distribution) of each species was obtained from occurrence data at the Global Biodiversity Information Facility (GBIF) database. We eliminated data from unknown sources, records without coordinates or with suspicious coordinates (i.e., in the ocean). We found a large variety of elevation distributions, from highly restricted species with ranges of 386 m, to species widely spread along different elevations with ranges of 4,800 m. Plant height was measured on 10 full grown individuals for each species in the study site from ground level to the tallest branch or leaf.

**Table 1:** *T*_*50*_ values, taxonomical and ecological information of the studied species.

Species	Family	Code	*T*_*50*_ (°C)	Elevation (m)	ΔElevation	Height (m)	Growth form
Paspalum hirtum	Poaceae	Pashir	45.4 ± 2.6	2600–3900	1300	0.09 ± 0.02	Grass
Bucquetia glutinosa	Melastomataceae	Bucglu	45.6 ± 0.9	2066–4104	2038	2.76 ± 0.67	Shrub
Orthrosanthus chimboracensis	Iridaceae	Ortchi	46.3 ± 2.7	1450–4000	2550	0.66 ± 0.08	Forbs
Aulonemia bogotensis	Poaceae	Aulbog	46.8 ± 3.4	3150–3625	475	0.78 ± 0.09	Grass
Valeriana pilosa	Caprifoliaceae	Valpil	47.2 ± 2.4	2600–3790	1190	0.29 ± 0.12	Forbs
Chusquea tessellata	Poaceae	Chutes	47.4 ± 1.8	1500–4350	2850	2.93 ± 1.29	Grass
Geranium multiceps	Geraniaceae	Germul	47.5 ± 2.0	2640–3600	960	0.24 ± 0.05	Forbs
Macleania rupestris	Ericaceae	Macrup	48.3 ± 0.8	600–4040	3440	1.95 ± 0.49	Shrub
Pentacalia vaccinioides	Asteraceae	Penvac	48.6 ± 1.0	2500–4700	2200	1.89 ± 0.34	Shrub
Lachemilla orbiculata	Rosaceae	Lacorb	48.6 ± 1.9	0–4800	4800	0.18 ± 0.01	Forbs
Oreopanax mutisianus	Araliaceae	Oremut	49.0 ± 2.0	2850–3600	750	2.20 ± 0.30	Shrub
Berberis goudotii	Berberidaceae	Bergou	49.0 ± 2.1	2640–4000	1360	2.24 ± 0.45	Shrub
Eryngium humboltii	Apiaceae	Eryhum	49.3 ± 0.6	1600–5600	4000	0.33 ± 0.08	Rosette
Pernettya prostrata	Ericaceae	Perpro	49.7 ± 1.1	640–4700	4060	0.80 ± 0.09	Shrub
Puya goudotiana	Bromeliaceae	Puygou	50.6 ± 2.7	3095–3481	386	1.63 ± 0.21	Rosette
Cortaderia columbiana	Poaceae	Corcol	51.1 ± 1.5	2000–4000	2000	1.23 ± 0.22	Grass
Espeletia grandiflora	Asteraceae	Espgra	51.3 ± 4.0	2640–4100	1460	1.77 ± 0.29	Rosette
Espeletia corymbosa	Asteraceae	Espcor	51.8 ± 3.1	2600–3724	1124	1.60 ± 0.14	Rosette
Espeletia argentea	Asteraceae	Esparg	52.4 ± 3.6	2600–3800	1200	0.58 ± 0.17	Rosette
Paepalanthus columbiensis	Eriocaulaceae	Paecol	52.9 ± 2.3	2600–3691	1091	0.33 ± 0.04	Rosette
Carex jamesonii	Cyperaceae	Carjam	53.9 ± 0.9	0–4100	4100	0.62 ± 0.14	Grass

List of species studied with name code used in the figures (Code), critical temperature (*T*_*50*_), elevation range of distribution (Elevation), delta of elevation (ΔElevation), plant height and growth form. Means and standard deviations are shown for height and *T*_*50*_.

### Heat tolerance–*T*_*50*_

To evaluate heat tolerance, we collected young fully expanded leaves from at least seven adult individuals per species. Collections and measurements were done between October 2016 and February 2018. We estimated *T*_*50*_ from the decline in *F*_*v*_*/F*_*m*_ observed on heated leaf disks using the following temperatures; 34°C, 38°C, 42°C, 48°C, 52°C, 56°C and 58°C. We followed Krause (2010) methodology. In short, *F*_*v*_*/F*_*m*_ was first recorded from non-heated disks to obtain the initial value for each species and to ensure sampled leaves were healthy. Then leaf disks were heated and kept for fifteen minutes at each temperature in a temperature-controlled water bath (Anova Precision Cooker, CA, USA). To prevent anaerobiosis, samples were placed inside a tea cloth bag, with one cloth layer in the adaxial side and three layers in the abaxial side of the leaf disks [46]. Disks enclosed in the tea cloth were placed in a zipped bag and into another watertight zipped bag containing a weight of 100 grams to ensure complete immersion of the leaf disks in the water bath and to avoid damping them. Control disks were not heated but enclosed in the bags for fifteen minutes at ambient temperature (21°C). Heated disks were then placed on petri dishes with wet paper towel and stored in the dark for 24h. Then, *F*_*v*_*/F*_*m*_ was recorded on these leaf disks with a modulated fluorometer OS30p+ (Opti-Sciences, Inc. NH, USA). We fitted a logistic curve on the change in *F*_*v*_*/F*_*m*_ with temperature for each individual and obtained the *T*_*50*_ as the temperature at which 50% reduction of initial *F*_*v*_*/F*_*m*_ value occurred. Curves were fitted with the “fitplc” R package, with the Weibull model and a 95% confidence interval modifying the “Kmax” argument so that it corresponded to the mean initial value for each species (all curves are in S2 Fig). The protocol is available at http://dx.doi.org/10.17504/protocols.io.29fgh3n [47].

### Thermal safety margin (TSM)

The TSM is often calculated as the difference between *T*_*50*_ and the mean air temperature, assuming equilibrium between leaf and air temperatures [31,48]. However, considering that leaf temperatures often exceed those of the air [41,46,48], here we calculated the TSM using the maximum daily leaf temperature (*T*_*max*_). *T*_*max*_ was obtained from thermal images taken with a portable thermal infrared camera Fluke Ti400 (Fluke corporation, WA, USA) on three mature leaves from seven individuals of each species between 10:00 a.m. until 2:00 p.m. only on sunny days between August and October 2018. We then calculated the thermal safety margins as *T*_*50*_ –*T*_*max*_.

### Statistical analysis

We ran two analyses to evaluate whether plant height and species elevation distribution could be used as proxies for heat tolerance. First, a multiple regression analysis evaluated the relationship between heat tolerance and the previously mentioned variables, also including maximum daily leaf temperature as an independent variable. Second, we ran a linear regression using a randomization analysis resampling the data for each continuous independent variable and fixing the predictor variables without replacement. Given the relatively small size of our sample, this second analysis also served to determine the slope of the relationship between each of the mentioned variables and to confirm the results of the multiple regression analysis.

To evaluate whether there were significant differences in *T*_*50*_ between growth forms, after controlling for the variance of the other continuous variables, we ran an ANCOVA. To account for the variation among species, we also ran a linear mixed model with growth form and leaf temperature as fixed effects and species as random term.

To evaluate whether species distribution, height or growth can explain the species thermal safety margins, we performed linear regressions between TSM and elevation, height and growth form. All statistical analyses were performed in R (R Development Core Team 3.4.2, r-project.org).

## Results

### Leaf and air temperatures

The monthly mean temperature at the study site during December 2017 to December 2018 was 8.3°C ± 0.7; the absolute minimum and maximum temperatures recorded were -2°C and 23.2°C respectively, both registered during the dry season that lasts from December until February (S1 Fig). As expected, values for leaf temperatures were much higher than those recorded for the air; *T*_*max*_ ranged between 21.3 and 33.3°C, demonstrating the decoupling from atmospheric conditions constantly mentioned in previous studies on alpine vegetation [30,38,49].

### Growth form, height, and elevation range

**Fig 1:**
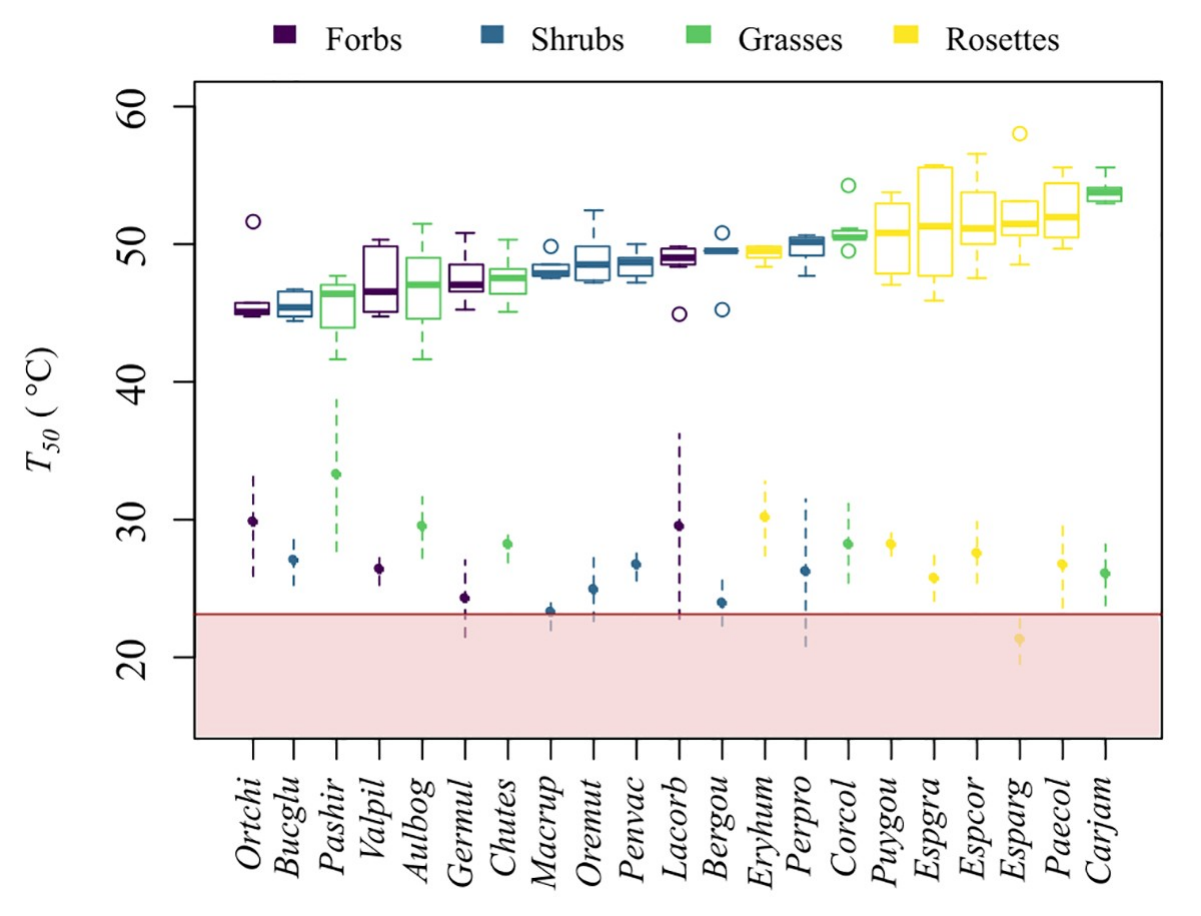
Heat tolerance (*T*_*50*_), maximum daily leaf temperature (*T*_*max*_) and maximum air temperature on site. Species *T*_*50*_ by growth form. Dots with dashed lines show the mean and standard deviation of *T*_*max*_. The red line indicates the maximum air temperature registered in the study site during the dry season of 2017–2018 (23.2°C), and the pink zone represents the range of current high air temperatures. Growth forms are depicted in different colors; purple represent forbs, blue shrubs, green grasses and yellow rosettes. The thermal safety margin is the differences between *T*_*50*_ and the pink zone.

The values of *T*_*50*_ ranged between 45.4°C and 53.9°C (Table 1, Fig 1), exceeding the maximum air temperature *in situ* by at least 22°C. The highest heat tolerance was found in rosettes (*p* = 0.047) with a *T*_*50*_ = 51.4 ± 1.30°C followed by grasses (*p* = 0.0013) with a *T*_*50*_ = 48.9 ± 3.49°C, and then by shrubs and forbs, both having similar values of heat tolerance (*p* = 0.920), and slightly lower than other growth forms. The mean *T*_*50*_ in shrubs was 48.4° ± 1.43°C, and in forbs was 47.4°C ± 0.95°C. For all studied species, the decrease in *F*_*v*_*/F*_*m*_ started at 42°C, but the rate of decline after this temperature differed between species (S2 Fig).

The best proxy for heat tolerance was growth form, which was found to be strongly correlated with *T*_*50*_ (F = 11.77, *p* = 7.51e-07), followed by the maximum daily leaf temperature *T*_*max*_ (F = 22.46, *p* = 5.69e-06). Elevation range and height were not good variables explaining heat tolerance (*p* = 0.203, *p* = 0.509 respectively; S3 Fig). The multiple regression analysis showed the same pattern, where the variation in *T*_*50*_ was poorly explained by those two variables (Adjusted *R*^*2*^ = 0.0354). Although, heat tolerance was negatively correlated with the maximum daily leaf temperature (*R*^*2*^ = 0.1522, *p* = 0.031), this relationship was driven only by grasses (S4 Fig). The linear mixed model confirms that growth form (*X* ^2^(1) = 6.45, *p* = 0.011), *T*_*max*_ (*X* ^2^(3) = 10.13, *p* = 0.017) and their interaction (*X* ^2^(3) = 10.87, *p* = 0.012) affects *T*_*50*_ values, even when species identity is included in the model.

### Thermal safety margin

**Fig 2:**
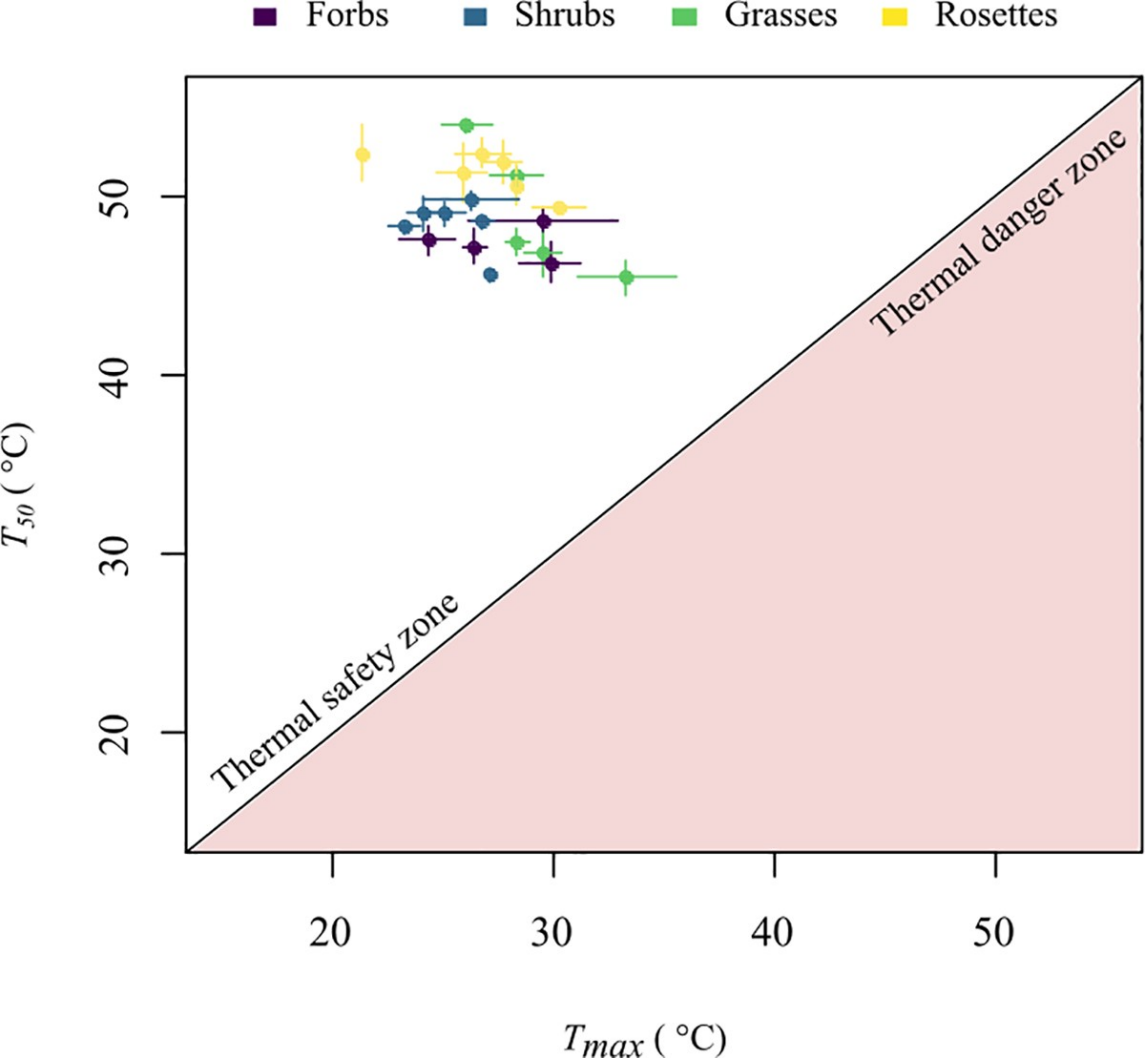
Visual representation of the thermal vulnerability for the 21 studied species. The diagonal line represents the zone where heat tolerance and leaf temperature values are the same, the white zone corresponds to the thermal safety zone or where *T*_*50*_ is higher than the maximum daily leaf temperature (*T*_*max*_), and the pink zone is the thermal danger zone where *T*_*50*_ is lower than *T*_*max*_. Colors represent the species’ growth form. Lines in each point are the standard error for both *T*_*50*_ and *T*_*max*_.

The critical temperature or *T*_*50*_ for all studied species was always much higher than *T*_*max*_ by at least 12 degrees; so, in general, species were far from experiencing temperatures near their critical maximum (Fig 2). Thermal safety margins calculated using the maximum daily leaf temperature ranged between 12.1°C and 31.0°C (S1 Table). The thermal safety margin was not related to elevation, growth form or height (F = 0.972, *p* = 0.465). Values of heat tolerance (*T*_*50*_) and the large difference between *T*_*50*_ and *T*_*max*_ suggests that all 21 paramo species are not physiologically vulnerable to increases in temperature and are within the thermal safety zone (Fig 2).

## Discussion

Our study assessed the heat tolerance (*T*_*50*_) and thermal safety margin of 21 species of the tropical alpine ecosystems (locally known as paramos) of the northern Andes. Values of heat tolerance were well above the highest air temperatures currently registered in the paramo, and above the highest leaf temperatures measured on site. The *T*_*50*_ or the critical temperature reflect an irreversible damage to the photosynthetic apparatus and correlates with membrane breakage, electrolyte leakage, necrosis of the leaves and a decline in net carbon assimilation [11,17,22]. In the 21 paramo plants we studied, these damages occurred at temperatures between 45.4°C to 53.9°C, temperatures that were 12.1°C to 31.0°C higher than today’s highest leaf temperature, and indicate a wide safety margin for paramo species. For the majority of the studied species, safety margins were broad enough to act as buffer for increases in temperature predicted for the RCP8.5 climate change scenario [50].

Contrary to our original expectation, we did not find evidence that highland specialists have lower heat tolerance than species with widespread distribution. Only the two extreme values supported this prediction; the species with the highest heat tolerance was a sedge, *Carex jamesonii*, with a widespread distribution from Mexico to Bolivia, found from sea level to 4,100 m, and the species with the lowest tolerance was a highland specialist, a grass, *Paspalum hirtum*, found only in Colombia and Ecuador restricted to 2,600–3,900 m in elevation. However, for the other 19 species, the values of heat tolerance were not related to their distribution, supporting the conservatism of heat tolerance previously mentioned [24,31,48]. Likewise we did not find evidence to support our second hypothesis that shorter plants should have higher heat tolerance than taller plants, a result expected if a thermal gradient between the ground and the air is generated and plants growing closer to the ground are exposed to more extreme thermal microhabitats [29,34]. In this Colombian Andean paramo we didn’t find evidence that leaf temperature on sunny days was higher in shorter plants than in taller plants, indicating that taller plants may warm their leaves by other means (i.e., insulating structures). The data does support our last hypothesis that, regardless of their height, rosettes have higher heat tolerance than other growth forms, suggesting that it is not height, but other traits of the rosettes that influences leaf temperature and heat tolerance.

Most studies of thermal tolerance in paramo plants to date have concentrated on the lower end of their thermal tolerance and have shown they have a remarkable capacity to withstand freezing temperatures. Our rosette group includes genera such as *Espeletia*, *Espeletiopsis* and *Paepalanthus*, which have insulating structures like densely pubescent leaves that also might bend inward at night to reduce heat loss to the surrounding air [51]. These species also retain their dead leaves around the stem to protect the stem pith from freezing [52,53] and resort to supercooling (the ability of plant tissues to maintain water in a liquid state at temperatures below the freezing point). These traits allow these species to easily withstand temperatures below 0°C and up to -16°C in some species [43,53,54].

However, some of these useful traits that support lower temperature tolerances could result in overheated leaves on sunny days [43] and therefore could require higher heat tolerance in these rosette species. Even though rosettes were generally better at tolerating high temperatures, all other growth forms (shrubs, forbs and grasses) had also quite high heat tolerance. Therefore, having a high heat tolerance in paramos might be a physiological adaptation to withstand the sudden variations in temperature, which ranges from freezing temperatures at night to high temperatures during clear days when high incoming solar radiation increases bare soil and plant surface temperature.

The values of heat tolerance we measured in paramo plants are within the range of values, from 35°C to 57°C, reported for tropical lowland woody species [31,42,55] and also within the range reported for temperate alpine species, that is within 47.4°C and 58°C [12,13]. The fact that heat tolerance is similar in the highlands and lowlands of the tropics and similar to values reported for the highlands of temperate zones supports the idea that tolerance to high temperature may be conserved across lineages and is poorly related to climatic metrics [24] as shown by Araujo et al. (2013) in a review of a large number of terrestrial ectotherms, endotherms and plants. In their review of 520 plant species, Araujo et al. (2013) show that the mean critical value of cold tolerance in plants is -20°C and the mean critical value of heat tolerance is 46°C, with a variance almost 24 times greater for cold tolerance than for heat tolerance.

The high heat tolerance we have found and the high cold tolerance reported for paramo plants in other studies [34–38] indicates that tropical alpine plants might have very broad ranges of thermal tolerance that helps them avoid or minimize leaf damage during freezing and during hot sunny days in the dry season when transpiration and leaf cooling may be compromised [44]. It therefore seems that temperature *per se* might not represent a serious threat to paramo plants. However, higher temperatures can also increase evaporative demand and decrease soil water availability with potential effects on plant-water relations and gas exchange [32,56]. This interaction needs to be explored further.

The fact that tolerance to high temperatures is highly conserved across clades in different ecosystems has positive implications for species from cold environments but potentially negative implications for species living in warm ecosystems. Data from 41 plants species from hot tropical dry forests consistently show these species are living closer to their thermal maxima. With apparently limited capacity to alter their upper thermal limits [31], these species are particularly vulnerable to ongoing climate warming. However, species from colder environments, such as paramo and alpine ecosystems that present high upper thermal limits well above current ambient temperature, are secured by a wide safety margin to physiologically withstand higher temperatures than what would be expected from their climatic niches. Data from these contrasting tropical ecosystems show that not all the tropical plants have similar thermal safety margins nor are all tropical plants already living very close to their optimal temperature as some studies suggest [15,22,57,58]. Our findings demonstrate that tropical species in the high mountains can tolerate warmer temperatures than the ones that occur within their current realized distributions, suggesting that rising temperatures will not necessarily result in range shifts [59,60] or potential extinction in paramo species, provided that heat tolerance plays a decisive role.

Usually the approach used to predict species' risks of extinction in the face of climate change integrates the species geographic ranges and climatic layers to evaluate changes on niche shifts. For tropical plant species, this approach is limited by the availability of reliable species occurrence and absence data, but also by the absence of more physiological studies to understand species’ ability to withstand warmer temperatures [61]. To make informed predictions about tropical alpine species’ fate in the face of climate change; we are in urgent need of more physiological studies and a better description of plant microclimate at a meaningful scale.

Our study only evaluated heat tolerance in adult plants, but studies on early developmental stages are lacking and could be vital for assessing vulnerability of paramo vegetation. Given that the highest temperatures are usually found near to the ground, especially on bare ground [8,62,63], seedlings are more at risk of heat injury [63] and their survival and establishment success will depend either on having high heat tolerance or on finding suitable conditions for safe recruitment [64,65]. To our knowledge there are no studies dealing with heat tolerance of early developmental stages of paramo plants, but in temperate alpine systems, seeds seem to be the most heat tolerant developmental stage, whereas seedlings are less tolerant and more prone to be lost by heat stress [63]. Seedling survival and recruitment in alpine areas could rely in the availability of suitable microclimates [7] typically protected among vegetation or stones [62,63]. In the Tropical Andes, several studies have shown that cushion plants, shrubs and even giant rosettes can function as nurse plants by changing microclimatic conditions, especially by reducing the prevalence of extreme temperatures and ameliorating the effects of drought [30,66–68], enhancing seedling survival and recruitment [62,69]. Additionally, small microtopographic variations can lead to substantial changes in soil temperature [8] and generate suitable sites for seedling recruitment. As long as safe sites are available, seedlings may be able to recruit, but more research is needed to understand species variation in heat tolerance on early stages of development in paramo plants.

Our data show that adult plants from paramo species have the physiological tolerance to withstand high temperatures and suggest that these species may not be living close to their upper thermal threshold. To better understand how climate change will affect paramo’s biodiversity, we still need to improve our ability to describe the thermal environment experienced by tropical alpine vegetation [7,8,70] in order to assess the physiological tolerance of adults and seedlings and to understand how temperature will affect other biotic interactions.

## Supporting information

S1 Fig Records of monthly air temperature on site from 2017 to 2018. Air temperature recorded with permanent sensors with solar radiation shields in the study site. The graph shows data for absolute maximum, monthly mean maximum, monthly mean, monthly mean minimum and absolute minimum temperatures during the two-year time (2017–2018). Red lines represent warmest temperatures while blue lines the coldest. The values 23.2°C and -2°C correspond to the warmest and coldest temperatures found during that year time.
(TIF)

S2 Fig Temperature response curve for *F_v_/F_m_* from where values of heat tolerance (*T_50_*) where obtained. Grey zone corresponds to the 95% confidence interval for the logistic fitted curve. Curves are organized by species from the lowest to the highest values of heat tolerance. Details for the species are provided in Table 1.
(TIF)

S3 Fig **Relationship between *T***_***50***_ **and delta of elevation (A), plant height (B) and *T***_***max***_ **(C).** Growth forms are depicted in different colors and each point represents the mean value of a species. The gray area shows the slopes for all linear regressions of the resampled data (n = 999) and the dark line shows the slope of the regression. The only significant relation was between heat tolerance and *T*_*max*_ (C; *R*^*2*^ = 0.1522; *p =* 0.031).
(TIF)

S4 Fig Linear regressions between *T_50_* and *T_max_* by growth forms. Each point represents an individual and the lines the slopes for the linear regressions. The ANCOVA analysis suggest a strong correlation between growth form and *T*_*max*_ (*p* = 0.00846), but most of this relationship was driven by the grasses (*p* = 0.00358).
(TIF)

S1 Table Thermal safety margins calculated from *T_max_* and *T_air_*. Mean values of *T*_*50*_ and *T*_*max*_ are shown with their respective standard deviations. Species are organized from lower to higher values of TSM_leaf_. We calculated TSM_air_ based on *T*_*air*_ as the absolute maximum air temperature recorded at the study site, that being 23.2°C.
(XLSX)
