# Supplementary figures and images for: High heat tolerance in plants from the Andean highlands: Implications for paramos in a warmer world
Source: PLoS One. 2019 Nov 6;14(11):e0224218. doi: 10.1371/journal.pone.0224218 (PMC6834248; doi:10.1371/journal.pone.0224218)

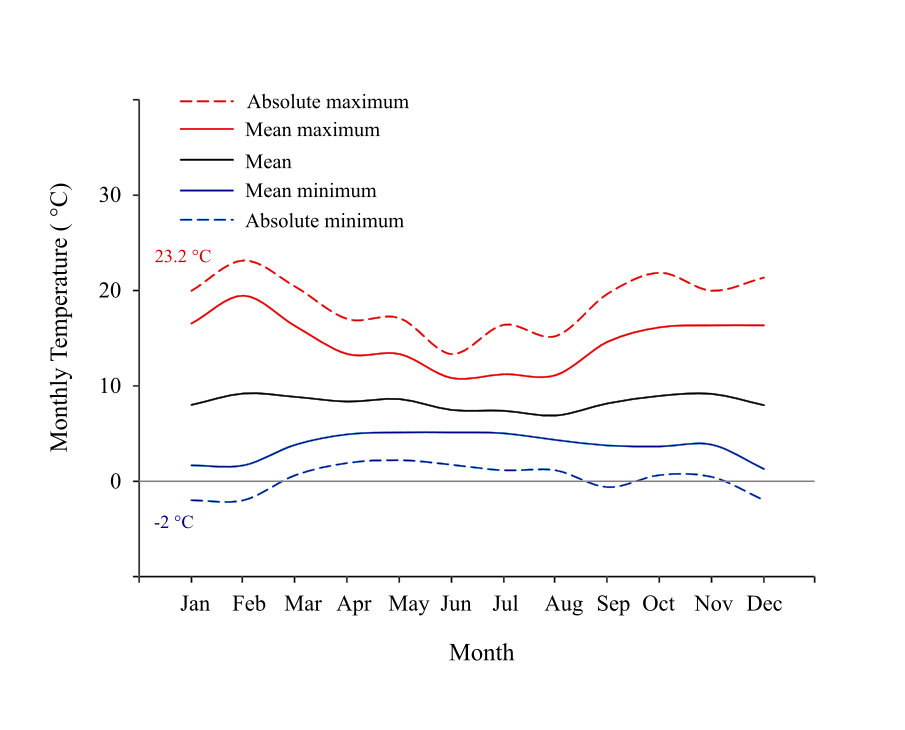

Supplement: S1 Fig — Air temperature recorded with permanent sensors with solar radiation shields in the study site. The graph shows data for absolute maximum, monthly mean maximum, monthly mean, monthly mean minimum and absolute minimum temperatures during the two-year time (2017–2018). Red lines represent warmest temperatures while blue lines the coldest. The values 23.2°C and -2°C correspond to the warmest and coldest temperatures found during that year time. (TIF) [file pone.0224218.s001.tif]

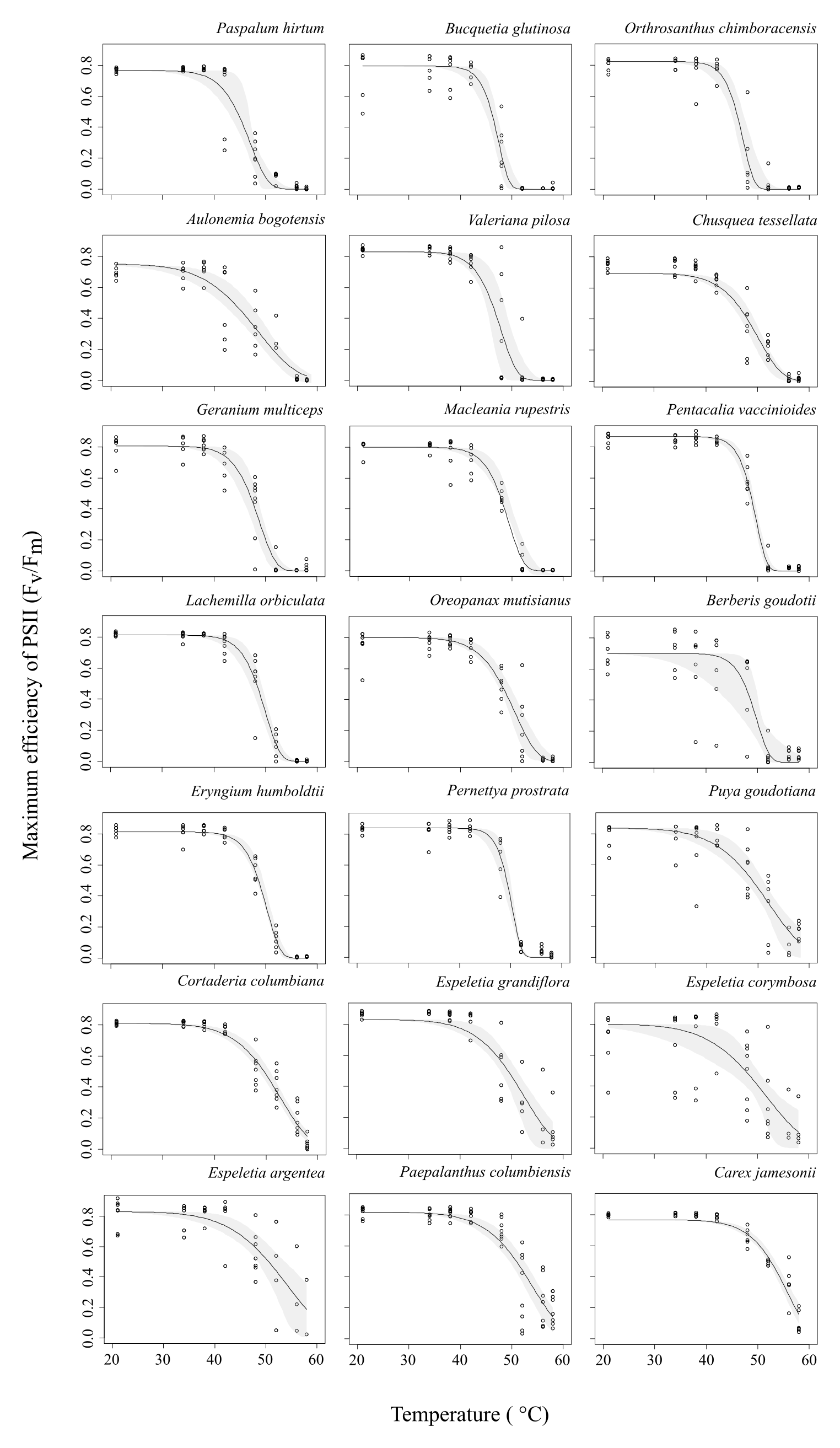

Supplement: S2 Fig — Grey zone corresponds to the 95% confidence interval for the logistic fitted curve. Curves are organized by species from the lowest to the highest values of heat tolerance. Details for the species are provided in Table 1. (TIF) [file pone.0224218.s002.tif]

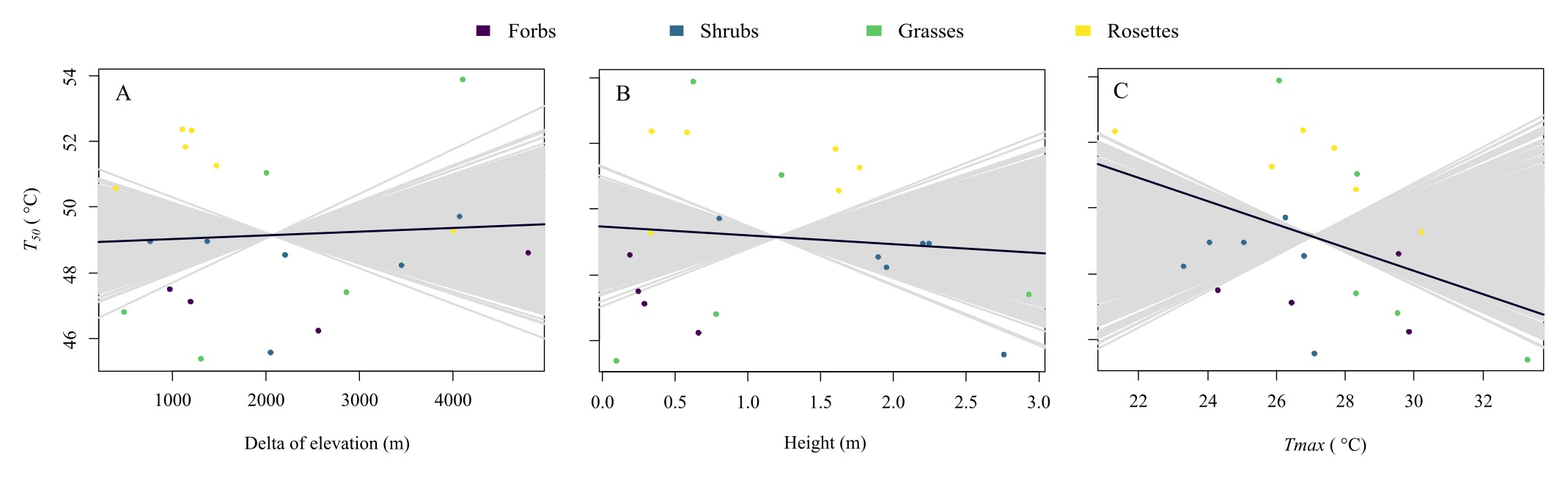

Supplement: S3 Fig — Relationship between T50 and delta of elevation (A), plant height (B) and Tmax (C). Growth forms are depicted in different colors and each point represents the mean value of a species. The gray area shows the slopes for all linear regressions of the resampled data (n = 999) and the dark line shows the slope of the regression. The only significant relation was between heat tolerance and Tmax (C; R2 = 0.1522; p = 0.031). (TIF) [file pone.0224218.s003.tif]

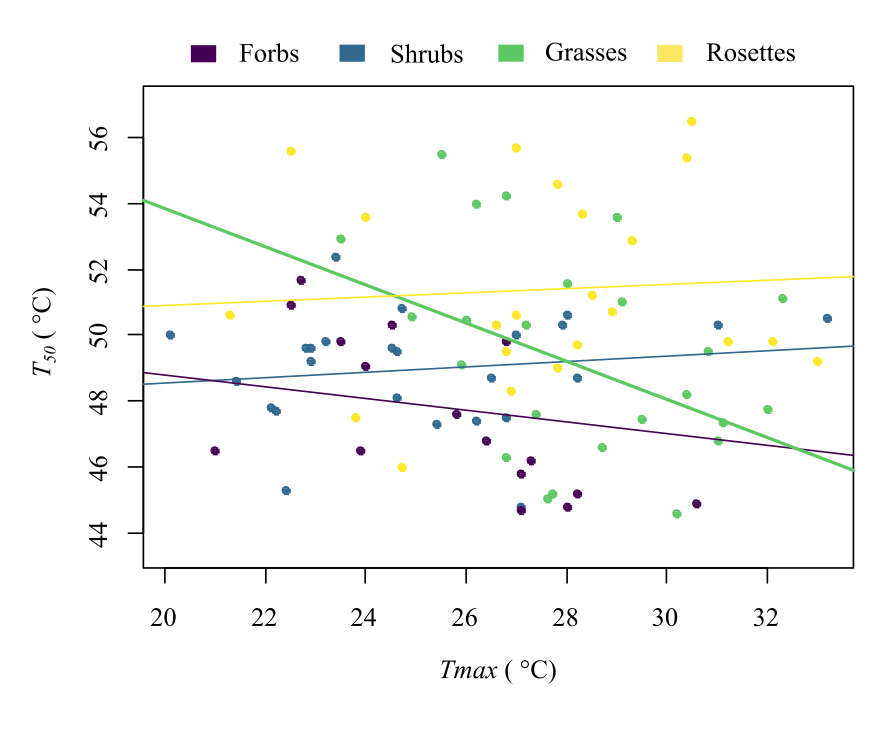

Supplement: S4 Fig — Each point represents an individual and the lines the slopes for the linear regressions. The ANCOVA analysis suggest a strong correlation between growth form and Tmax (p = 0.00846), but most of this relationship was driven by the grasses (p = 0.00358). (TIF) [file pone.0224218.s004.tif]
